# A viral vector prime and protein boost vaccine regimen elicits higher binding antibody titers than a homologous viral vector prime-boost regimen

**DOI:** 10.1093/immhor/vlaf027

**Published:** 2025-10-30

**Authors:** Bakare Awakoaiye, Tanushree Dangi, Pablo Penaloza-MacMaster

**Affiliations:** Department of Microbiology-Immunology, Feinberg School of Medicine, Northwestern University, Chicago, IL, United States; Department of Microbiology-Immunology, Feinberg School of Medicine, Northwestern University, Chicago, IL, United States; Department of Microbiology-Immunology, Feinberg School of Medicine, Northwestern University, Chicago, IL, United States; Center for Virology and Vaccine Research, Beth Israel Deaconess Medical Center, Boston, MA 02215, United States

**Keywords:** antibodies, vaccines, viral immunity

## Abstract

(SARS-CoV-2) has infected a large fraction of the human population. Currently, most individuals have developed immunity either through vaccination or natural infection. Despite this, SARS-CoV-2 booster immunizations are still recommended to reduce the risk of reinfections, but there is still limited understanding of how different booster vaccine platforms influence antibody responses. We conducted immunological studies in mice to evaluate the boosting effects of different vaccine platforms on antibody responses. C57BL/6 mice were first primed with an adenovirus serotype 5 (Ad5) vector vaccine expressing the SARS-CoV-2 spike protein. The mice were then boosted with the same Ad5-based vaccine (homologous boosting) or with a protein-based vaccine (heterologous boosting). Interestingly, the heterologous regimen (Ad5 prime/Protein boost) elicited higher binding antibody responses, relative to the homologous regimen (Ad5 prime/Ad5 boost). Similar potentiation of antibody titers was reported when mice were primed with poxvirus or rhabdovirus vectors and then boosted with protein. These findings highlight a potential advantage of protein booster immunizations to potentiate humoral immunity.

## Introduction

The coronavirus disease 2019 (COVID-19) pandemic has led to widespread global immunity to severe acute respiratory syndrome coronavirus 2 (SARS-CoV-2) through vaccination, natural infection, or a combination of both. However, waning antibody responses over time have been linked to an increased risk of breakthrough infections, underscoring the importance of booster vaccinations to sustain protective immunity.^1^ Despite the broad deployment of booster vaccines, there is still limited understanding of how different vaccine platforms influence antibody responses after boosting.

Adenovirus-based vaccines, including Ad5-based vaccines such as the CanSino and Sputnik V vaccines, and vaccines utilizing other adenovirus serotypes like Ad26 or chimpanzee adenovirus, have been administered to millions of people worldwide and demonstrated efficacy against COVID-19.^2–4^ Additionally, mRNA vaccines and protein subunit vaccines, which are key components of the global vaccination effort, have played a significant role in curbing the pandemic and are currently authorized for use in many countries, including the United States. In particular, protein vaccines offer the advantage of low reactogenicity compared to other vaccine platforms, making them an appealing option for booster immunizations.

Given the global use of multiple vaccine platforms during the COVID-19 pandemic, including adenovirus vectors, a key question is how different combinations of these platforms influence immune outcomes. Heterologous prime-boost strategies, where the priming and boosting vaccines are from different platforms, have gained attention for their potential to enhance immune responses. However, in human populations, it remains challenging to dissect the individual contributions of the priming and boosting components due to variability in infection history, vaccination schedules, and host factors. To address these complexities, we turned to a controlled animal model that allows for systematic evaluation of vaccine platform combinations.

To further explore how protein booster vaccines affect immune responses, we conducted studies in mice primed with viral vector vaccines. These mice were subsequently boosted either with homologous viral vector vaccines or with heterologous protein vaccines. Our findings reveal that heterologous boosting with protein vaccines elicits significantly higher binding antibody titers compared to homologous viral vector boosting. These results provide insights into optimizing vaccine booster strategies to specifically enhance humoral immunity.

## Results

### Priming with Ad5 and boosting with protein elicits higher SARS-CoV-2 specific antibody titers, relative to priming and boosting with Ad5

**Figure 1.**
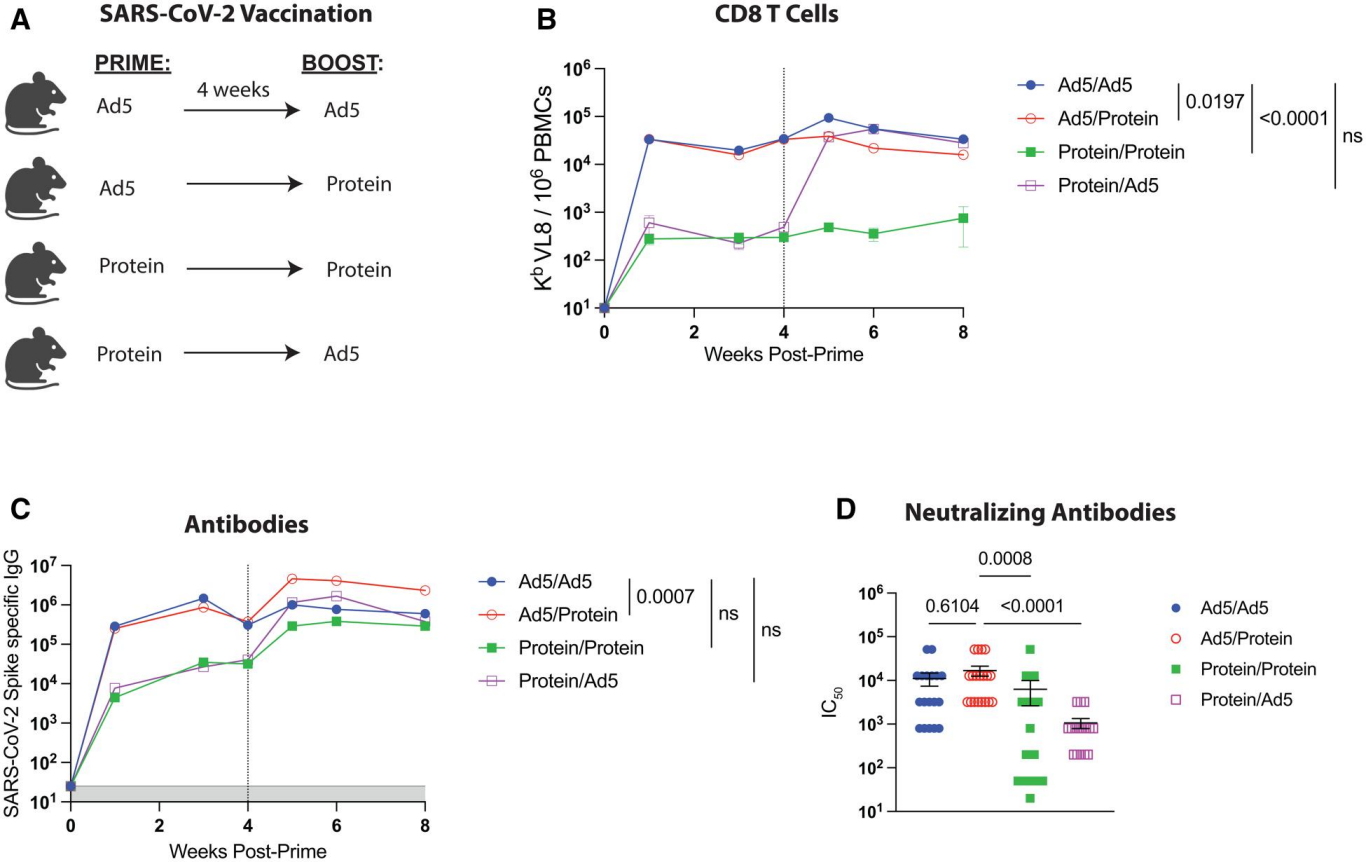
Priming with Ad5-SARS-CoV-2 spike and boosting with SARS-CoV-2 spike protein elicits higher antibody titers, compared to homologous prime-boost vaccine regimen. (A) Experimental approach for evaluating how protein boosting affects adaptive immune responses. C57BL/6 mice were primed and boosted with either 10^8^ PFU of an Ad5-SARS-CoV-2 spike vaccine, or 10 µg of spike protein in 1:10 Adju-Phos. (B) Summary of SARS-CoV-2-specific CD8^+^ T cell titers (K^b^ VL8+) in PBMCs. (C) Summary of SARS-CoV-2-specific antibody titers in sera. (D) Summary of SARS-CoV-2–neutralizing antibody responses in sera at day 7 post-boost. IC_50_, median inhibitory concentration. Data from panels A-C are from five experiments, with *n* = 3-7 mice per group. The vertical dashed line indicates the time of boosting. Data from panel D are from four experiments, with *n* = 3-5 mice per group. All data are shown. Indicated *P* values were determined by one-way ANOVA Kruskal-Wallis test with Dunn's multiple comparisons. Error bars represent SEM.

We first primed C57BL/6 mice intramuscularly with an Ad5 adenovirus vector expressing SARS-CoV-2 spike protein (Ad5-SARS-CoV-2 spike) used in prior studies^5^ or a spike protein vaccine. After approximately four weeks, mice were boosted homologously or heterologously, and immune responses were measured in blood (Fig. 1A). Priming and boosting with the same Ad5 vaccine resulted in an increased number of antigen-specific CD8 T cells, relative to Ad5 priming/Protein boosting, exhibiting a 2-fold difference between the two groups (Fig. 1B). However, Ad5 priming/Protein boosting resulted in a 3.9-fold superior difference in binding antibody titers when compared to priming and boosting with Ad5 (Fig. 1C). We also observed a similar pattern of increased neutralizing antibody titers with the Ad5 prime/Protein boost group, but it was not statistically significant (Fig. 1D).

**Figure 2.**
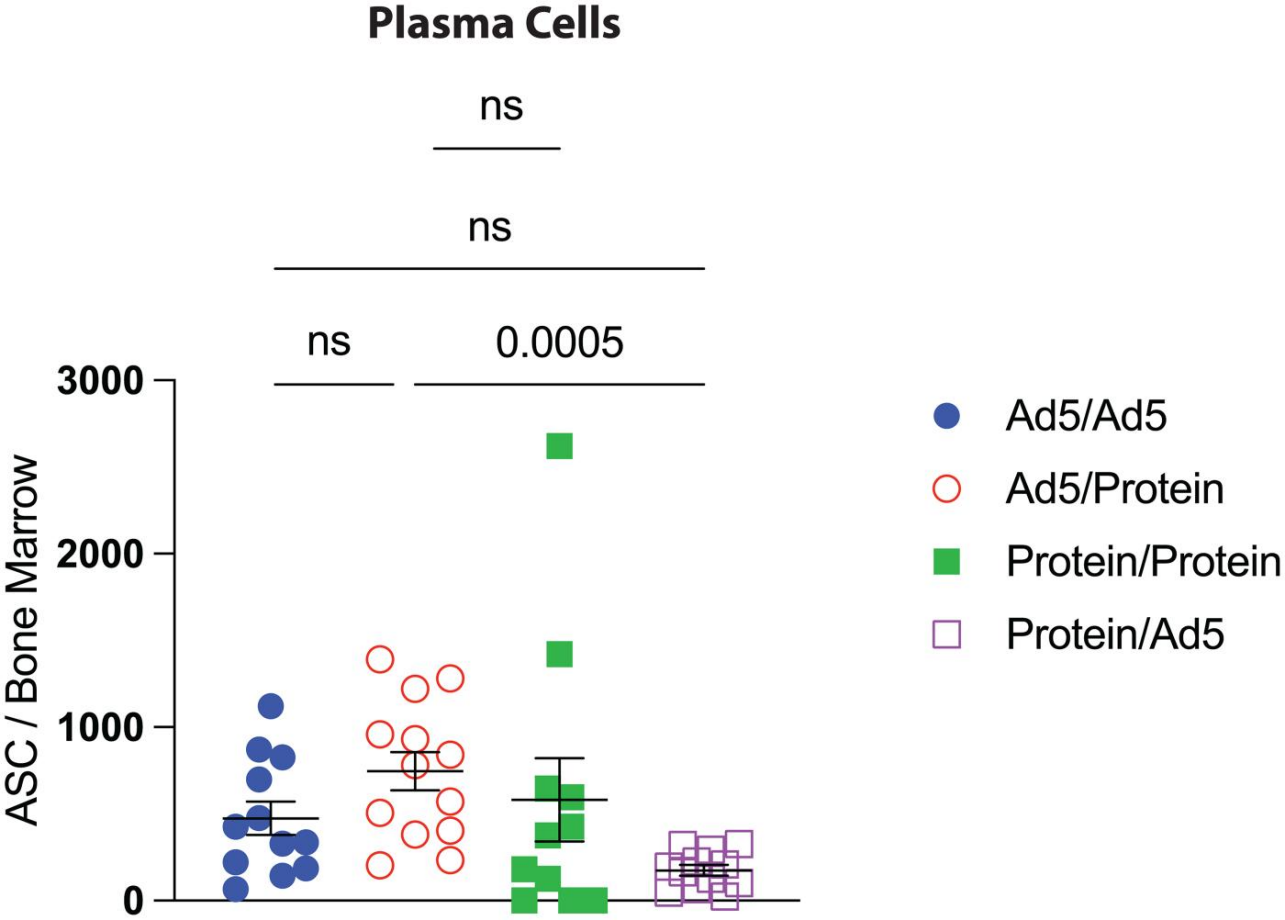
Plasma cells following vaccination. C57BL/6 mice were primed and boosted with either 10^8^ PFU of an Ad5-SARS-CoV-2 spike vaccine, or 10 µg of spike protein in 1:10 Adju-Phos. SARS-CoV-2-specific antibody-secreting cells were enumerated in bone marrow after 20 weeks. Data are from 3 experiments, with *n* = 3-5 mice per group. Each experiment included 1-2 naive control mice. All data are shown. Indicated *P* values were determined by 1-way ANOVA Kruskal–Wallis test with Dunn's multiple comparisons. Error bars represent SEM.

As plasma cells are a primary source of antibody production, we investigated if these antibody titer differences were associated with plasma cell numbers. We found that the enhancement of antibody titers observed in the heterologous prime-boost regimen was associated with an increase in plasma cells in the bone marrow (Fig. 2). The Ad5/Protein group showed a pattern of enhanced responses relative to other groups, but the difference was only statistically significant when comparing the Ad5/Protein group to the Protein/Ad5 group.

### Generalizability to other Ad5 vaccines

**Figure 3.**
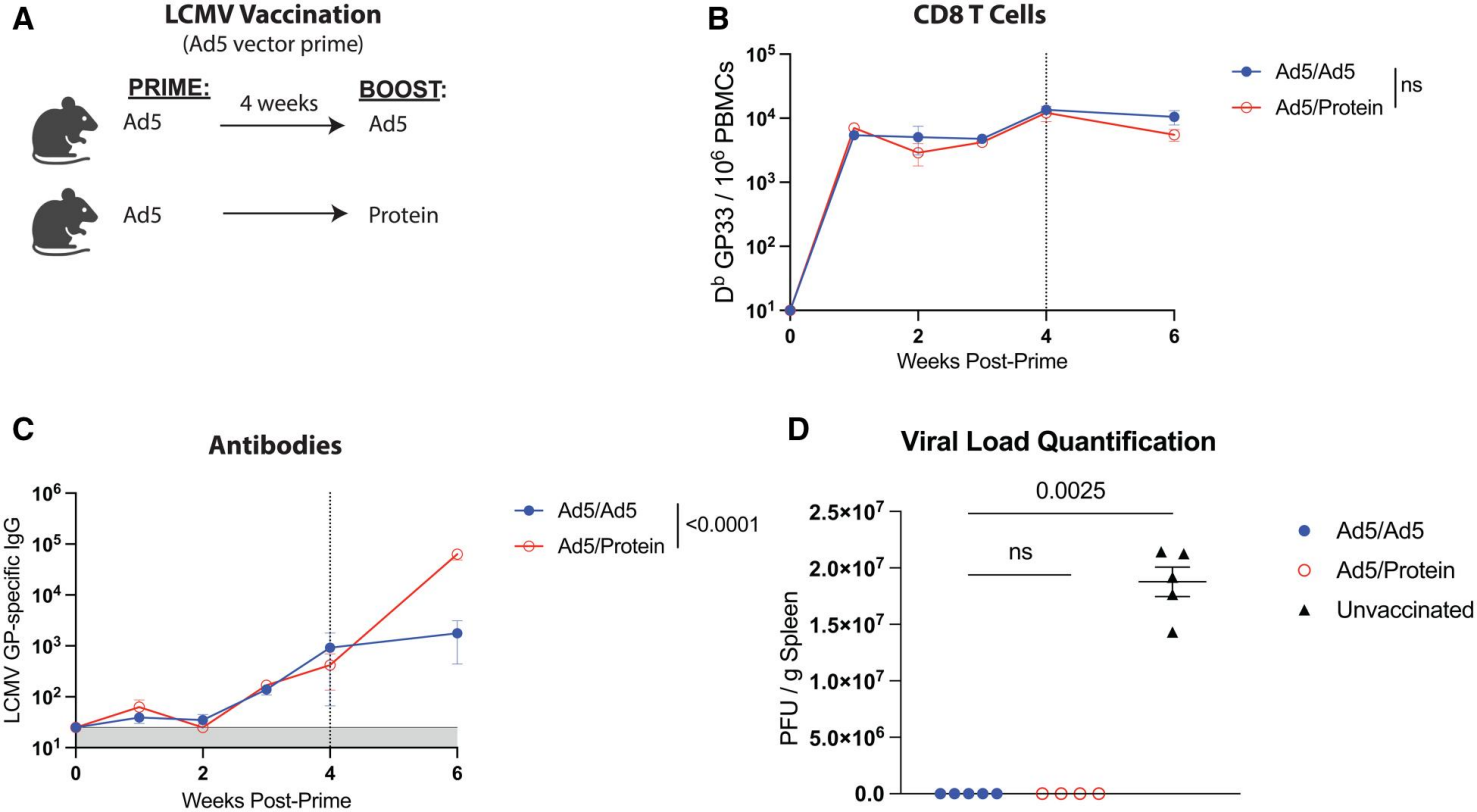
Priming with Ad5-LCMV GP and boosting with LCMV GP protein elicits higher antibody titers, compared to homologous prime-boost vaccine regimen. (A) Experimental approach for evaluating how protein boosting affects adaptive immune responses. C57BL/6 mice were primed and boosted with either 10^8^ PFU of an Ad5-LCMV GP vaccine, or 10 µg of GP protein in 1:10 Adju-Phos. (B) Summary of LCMV-specific CD8^+^ T cell titers (D^b^ GP33+) in PBMCs. (C) Summary of LCMV-specific antibody titers in sera. (D) Spleen viral loads at day 7 after LCMV Cl-13 challenge (intravenous) quantified by plaque assays on Vero cell monolayers. Data from panels A to C are from 3 experiments, with *n* = 4-5 mice per group. The vertical dashed line indicates the time of boosting. Data from panel D are from one experiment, with *n* = 4-5 mice per group. All data are shown. Indicated *P* values were determined by Mann–Whitney test or 1-way ANOVA Kruskal-Wallis test with Dunn's multiple comparisons. Error bars represent SEM.

The above studies only involved Ad5 and protein vaccines for SARS-CoV-2, so we broadened our analysis to determine whether our findings could generalize to other antigens. We primed C57BL/6 mice intramuscularly with an Ad5 adenovirus vector expressing lymphocytic choriomeningitis virus (LCMV) glycoprotein (Ad5-LCMV GP). After approximately four weeks, mice were boosted homologously with Ad5-LCMV GP or heterologously with the respective protein, LCMV GP, and immune responses were measured in the blood (Fig. 3A). Priming and boosting with the same Ad5 vaccine resulted in similar numbers of LCMV-specific CD8 T cells, relative to Ad5 priming/Protein boosting (1.9-fold difference but not statistically significant) (Fig. 3B). However, the heterologous Ad5 prime/Protein boost resulted in a 36-fold superior difference in LCMV-specific binding antibody titers when compared to priming and boosting with Ad5 (Fig. 3C). Additionally, we performed viral challenges with chronic LCMV Cl-13. We compared viral loads in the spleen measured by plaque assays, but all vaccinated mice cleared the virus, and we could not discern differences in viral control between the 2 groups (Fig. 3D). This is because the level of memory immune responses necessary to clear an LCMV Cl-13 challenge is very low,^6^ making it difficult to ascertain differences in immune protection by our vaccine regimens.

### Generalizability to other vaccine platforms

**Figure 4.**
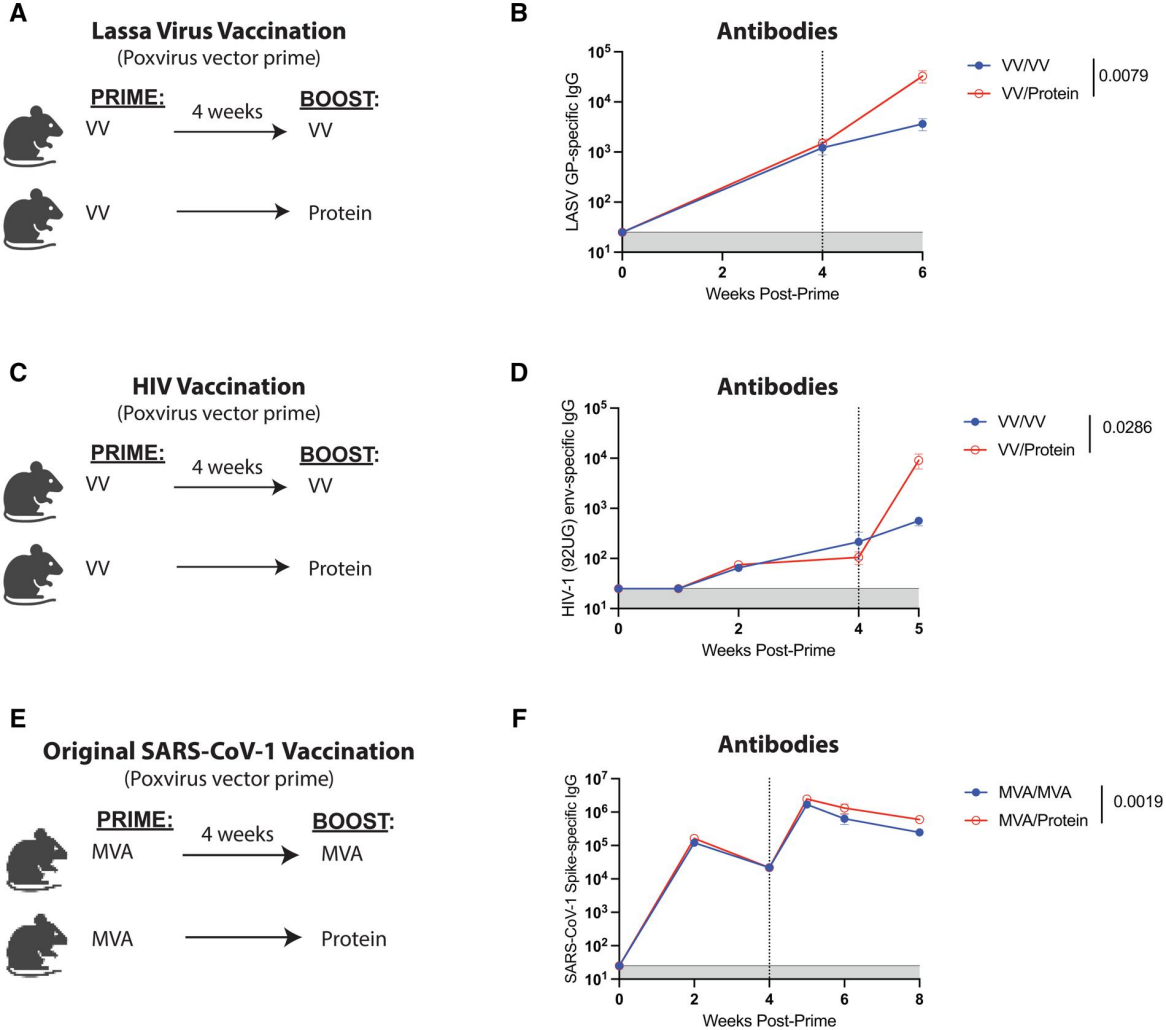
Priming with poxvirus vectors and boosting with protein elicits higher antibody titers. (A) Experimental approach. C57BL/6 mice were primed and boosted with either 10^8^ PFU of a VV-Lassa GP vaccine, or 5 µg of Lassa GP protein in 1:10 Adju-Phos. (B) Summary of Lassa-specific antibody titers in sera. (C) Experimental approach. C57BL/6 mice were primed and boosted with either 7 × 10^7^ PFU of a VV-HIV Env (strain 92UG) vaccine, or 100 µg of HIV Env protein in 1:10 Adju-Phos. (D) Summary of HIV-specific antibody titers in sera. (E) Experimental approach. C57BL/6 mice were primed and boosted with either 3 × 10^6^ PFU of an MVA-SARS-CoV-1 vaccine, or 10 µg of SARS-CoV-1-spike in 1:10 Adju-Phos. (F) Summary of SARS-CoV-1-specific antibody titer in sera. Data from panels B and D are from 1 experiment, with *n* = 4-5 mice per group. Data from panel F are from 2 experiments, with *n* = 4-5 mice per group. The vertical dashed line indicates the time of boosting. All data are shown. Indicated *P* values were determined by Mann–Whitney test. Error bars represent SEM.

The above studies involved Ad5 and protein vaccines expressing various antigens, and we then explored whether our findings could generalize to different viral vector platforms other than Ad5. We primed C57BL/6 mice intramuscularly with a poxvirus vector (vaccinia virus) expressing Lassa virus glycoprotein (VV-Lassa GP). After 4 wk, mice were boosted homologously with VV-Lassa GP or heterologously with the respective protein, Lassa GP, and antibody responses were measured in the blood (Fig. 4A). Priming with VV-Lassa GP and boosting with protein resulted in a 9-fold difference in binding antibody titers, relative to the homologous VV-Lassa GP prime-boost regimen (Fig. 4B). Similar patterns were observed with a vaccinia virus vector expressing a human immunodeficiency virus type 1 (HIV-1) envelope protein (VV-HIV env) (16-fold difference) (Fig. 4C, D), and a modified vaccinia virus Ankara (MVA) expressing SARS-CoV-1 spike protein (MVA-SARS-CoV-1 spike) (2.4-fold difference) (Fig. 4E, F).

**Figure 5.**
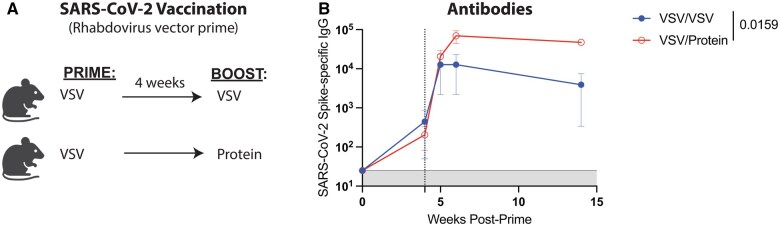
Priming with a rhabdovirus vector and boosting with protein elicits higher antibody titers. (A) Experimental approach. C57BL/6 mice were primed and boosted with either 6 × 10^5^ PFU of a VSV-SARS-CoV-2 spike vaccine, or 10 µg of SARS-CoV-2 spike protein in 1:10 Adju-Phos. (B) Summary of SARS-CoV-2-specific antibody titers in sera. Data are from one experiment, with n = 5 mice per group. The vertical dashed line indicates the time of boosting. All data are shown. Indicated *P* values were determined by Mann–Whitney test. Error bars represent SEM.

In addition, we investigated whether our findings could extend to a vesicular stomatitis virus vector (VSV) expressing a SARS-CoV-2 spike protein (VSV-SARS-CoV-2 spike). We first primed C57BL/6 mice intramuscularly with VSV-SARS-CoV-2 spike, and after about four weeks, mice were boosted homologously with the same VSV-SARS-CoV-2 spike viral vector vaccine or heterologously with SARS-CoV-2 spike protein, and antibody responses were measured in blood. Priming with the VSV-SARS-CoV-2 spike vaccine and boosting with the spike protein vaccine resulted in a 12-fold difference in binding antibody titers, relative to the homologous prime-boost regimen (Fig. 5A, B). Altogether, these findings suggest that protein boosters are particularly useful to enhance binding antibody titers, regardless of viral vector or target antigen.

## Discussion

Although SARS-CoV-2 vaccines prevent severe disease and death, they do not confer durable sterilizing immunity due in part to viral variants as well as waning antibody titers, warranting the use of boosters. In this report, we show that a heterologous regimen composed of a viral vector prime followed by a protein boost results in higher antibody titers compared to a homologous prime-boost with viral vectors. What is the mechanistic basis for this? It is possible that after a viral vector prime, Ad5-specific antibodies generated by prior infection with Ad5 or related adenoviruses could reduce the boosting potential of Ad5. Pre-existing immunity elicited by either active or passive immunization can affect the boosting capacity of vaccines,^7^^,^^8^ and in particular, anti-vector immunity is known to accelerate Ad5 vaccine clearance and immunogenicity of viral vectors like Ad5.^9^ It is also possible that soluble protein is more effective at engaging B cell receptors (BCR) on the surface of memory B cells. Another important aspect to highlight in some of our vaccine studies (SARS-CoV-2 vaccination studies), is that the homologous Ad5 prime/Ad5 boost regimen tends to elicit slightly greater numbers of CD8 T cells than the heterologous Ad5 prime/Protein boost regimen. This is likely because viral vectors express their antigens intracellularly, favoring CD8 T cell responses. This bias toward T cell responses becomes more evident when comparing the homologous Ad5 group to the homologous Protein group. We observed a 45-fold difference, further validating that viral vectors induce significantly greater T cell responses than protein vaccines. Interestingly, in the case of Ad5-LCMV GP, the difference in LCMV-specific CD8 T cell populations between the homologous Ad5 regimen and the heterologous Ad5 prime/Protein boost was not statistically significant.

Our data with Ad5 vectors expressing different antigens (Ad5-SARS-CoV-2 spike and Ad5-LCMV GP) and our data with different viral vector platforms (VV, MVA, VSV) suggest generalizability. We did not conduct SARS-CoV-2 challenge experiments to compare immune protection because in prior mouse studies, vaccinating with a single dose of the Ad5-SARS-CoV-2 spike vaccine conferred robust protection, making it difficult to detect differences in immune protection between homologous and heterologous Ad5 vaccine regimens. High levels of immune protection have also been described with other SARS-CoV-2 vaccines based on adenoviruses.^10–14^ Similarly, a single dose of Ad5-LCMV GP vaccine has been shown to result in very rapid clearance of chronic LCMV Cl-13.^6^ We also observed this in our studies, as we could not discern differences in LCMV Cl-13 viral control between the homologous Ad5 primed/Ad5 boosted group and the heterologous Ad5 primed/Protein boosted group. Altogether, the LCMV Cl-13 model does not have the necessary resolution to ascertain differences in viral control between the different vaccine regimens. Future studies will address whether the enhanced binding antibody titers of the heterologous regimen could provide enhanced protection against other pathogens.

It is possible that a viral vector prime and protein boost regimen may be useful in individuals who develop suboptimal antibody responses following vaccination, including the elderly. Protein boosters may also be especially effective in the context of HIV vaccination, where antibody responses are believed to be more critical than T cell responses at preventing initial infection and the establishment of the viral reservoir. Boosting with protein may also improve uptake and safety in the population, given that protein vaccines typically induce lower adverse events compared to other vaccine platforms.

We also study antibody responses against a variety of antigens, not only SARS-CoV-2, but also other pathogens like HIV, Lassa virus (LASV), and LCMV. While there are now numerous vaccines for SARS-CoV-2, there are no approved vaccines for HIV. Some of the biggest challenges for developing an HIV vaccine are the high degree of viral variability and the difficulty in generating broadly neutralizing antibodies against its highly glycosylated envelope glycoprotein. Interestingly, the RV144 HIV-1 vaccine trial consisting of a viral vector prime, protein boost regimen showed a moderate, but significant, level of protection against HIV.^15^ LASV and LCMV are arenaviruses with extensive genetic diversity and are associated with poor outcomes among pregnant and immunocompromised individuals. LASV is a leading cause of hemorrhagic fever and the WHO has designated LASV as a priority pathogen, indicating the need for the development of a vaccine.^16^ LCMV, on the other hand, has been more thoroughly investigated as a model to study viral immunology, so there has not been much impetus for developing vaccines, despite this virus being a rising concern for transplant patients.

It is also important to note that while only a small fraction of people in the United States received adenovirus vaccines during the COVID-19 pandemic, these vaccines served as the primary option for many underdeveloped countries. Future studies will determine whether protein boosters have the same effect in the setting of mRNA vaccines. Altogether, these data suggest that protein boosters favor the elicitation of potent antibody responses in hosts that were previously primed with viral vectors, providing insights for rational vaccine design.

## Materials and methods

### Vaccines

We used a non-replicating Ad5 vector that is E1/E3 deleted and expresses SARS-CoV-2 spike protein (strain 2019-nCoV-WIV04) within the putative E1 site.^5^^,^^17^ This vector contains a Cytomegalovirus (CMV) promoter driving the expression of the SARS-CoV-2 spike protein and was used in prior papers.^5^^,^^9^ The other Ad5 vectors have a similar genetic profile, but they express their respective transgene in the putative E1 site, instead of spike. The Ad5 vectors were propagated at the Iowa Vector Core on trans-complementing HEK293 cells (ATCC), purified by cesium chloride density gradient centrifugation, titrated, and then frozen at −80°C. All the other vectors were propagated in-house. The poxvirus vectors (VV and MVA) were a kind gift from Dr. Bernard Moss (NIH). The rhabdovirus vector (VSV) was a kind gift from Dr. Sean Whelan (Washington University in St Louis). The Ad5-LCMV GP vector was a kind gift from Dr. Julie McElrath (Fred Hutchinson Cancer Research Center).

SARS-CoV-2 spike protein was made in-house using a plasmid that was produced under HHSN272201400008C and obtained through Biodefense and Emerging Infections (BEI) Resources, National Institute of Allergy and Infectious Diseases (NIAID), National Institutes of Health (NIH): Vector pCAGGS containing the SARS-Related Coronavirus 2, Wuhan-Hu-1 Spike Glycoprotein Gene (soluble, stabilized), NR-52394. We used a HEK293T mammalian cell expression system to express spike protein. Cell culture supernatant was collected and the expressed protein within this supernatant was purified by fast protein liquid chromatography (FPLC) followed by dialysis. Quality and purity (>95%) were checked in SDS-PAGE (140-180 kDa size under reducing conditions) and in western blot.

SARS-CoV-1 spike protein was obtained through BEI Resources, NIAID, NIH: SARS-CoV Spike (S) Protein deltaTM, Recombinant from Baculovirus, NR-722. Lassa Virus Glycoprotein was obtained through BEI Resources, NIAID, NIH: Glycoprotein from Lassa Virus, ISTH-2018-014, Recombinant from Baculovirus, NR-51469. HIV-1 Env gp140 protein (Clade A, 92UG037) was a kind gift of Dr. James Kovacs, who used a HEK293T mammalian cell expression system. A hybridoma for expressing LCMV GP Cl-13 protein was a kind gift of Drs. Carl Davis and Rafi Ahmed. The same proteins used as vaccines were also used as coating antigens for the ELISA and ASC assays.

### Mice and vaccinations

In brief, 6- to 8-wk-old C57BL/6 mice were used. Mice were purchased from Jackson laboratories (approximately half males and half females). Mice were immunized intramuscularly (50 µl per quadriceps) with the respective vaccine diluted in sterile phosphate-buffered saline (PBS). Mice were housed at the Northwestern University Center for Comparative Medicine (CCM) in downtown Chicago. All mouse experiments were performed with approval from the Northwestern University Institutional Animal Care and Use Committee (IACUC).

### Reagents, flow cytometry, and equipment

Single-cell suspensions were obtained from PBMCs and various tissues as described previously.^18^ Dead cells were gated out using Live/Dead fixable dead cell stain (Invitrogen). Biotinylated MHC class I monomers (K^b^ VL8, sequence VNFNFNGL from the spike protein of SARS-CoV-2, and D^b^GP33, sequence KAVYNFATC from the GP of LCMV, used and tested in prior studies^19–24^) were obtained from the NIH tetramer facility at Emory University and were tetramerized in house. Cells were stained with fluorescently labeled antibodies against CD8α (53-6.7 on PerCP-Cy5.5) purchased from BD Pharmingen and CD44 (IM7 on Pacific Blue) purchased from BioLegend. Flow cytometry samples were acquired with a Becton Dickinson Canto II or an LSRII and analyzed using FlowJo (Treestar).

### ELISA

Binding antibody titers were measured using ELISA as described previously,^25^ but using protein instead of viral lysates. In brief, 96-well flat bottom plates MaxiSorp (Thermo Scientific) were coated with 0.1 μg/well of the respective protein, for 48 h at 4°C. Plates were washed with PBS + 0.05% Tween-20. Blocking was performed for 4 hr at room temperature with 200 μl of PBS + 0.05% Tween-20 + bovine serum albumin. 6 μl of sera were added to 144 μl of blocking solution in the first column of the plate, 1:3 serial dilutions were performed until row 12 for each sample, and plates were incubated for 60 min at room temperature. Plates were washed three times followed by the addition of horseradish peroxidase (HRP)-conjugated goat anti-mouse IgG (Southern Biotech) diluted in blocking solution (1:1,000), at 100 μl/well and incubated for 60 minutes at room temperature. Plates were washed three times and 100 μl/well of Sure Blue substrate (Sera Care) was added for approximately 8 minutes. The reaction was stopped using 100 μl/well of KPL TMB stop solution (Sera Care). Absorbance was measured at 450 nm using a Spectramax Plus 384 (Molecular Devices).

### B cell ELISPOT assay

For detecting antibody-secreting cells (ASC) we used a protocol from a prior publication, but we used spike protein as the coating antigen instead of viral lysate.^25^ In brief, SARS-CoV-2 spike-specific ASCs were quantitated by ELISPOT using a 96-well Multiscreen filter plate (MSHAN4B50, Millipore Ireland BV). Plates were coated with 0.5 μg/well of the SARS-CoV-2 spike protein and incubated overnight at 4°C. After incubation, plates were washed once with PBS containing 0.05% Tween 20 (PBS-T) and twice with PBS. Plates were blocked by incubating plates with RPMI containing 10% fetal bovine serum (FBS) for 2 h at room temperature. Single suspensions of bone marrow cells at 60x10^6^ cells/mL were prepared in medium (RPMI supplemented with 10% FBS, 1% penicillin/streptomycin, and 1% L-glutamine and 0.05 mM of B-Mercaptoethanol). After incubation, the blocking medium was replaced with 100 μl/well of fresh medium, and 50 μl of single-cell suspension was added to the first row and serially diluted 3-fold down the plate. Plates were incubated for 8 h at 37°C in a 5% CO_2_ incubator. Plates were washed with PBS and PBS-T, and incubated with 100 μl of biotinylated anti-mouse IgG antibody diluted 1:1,000 in PBS-T with 1% FBS for two days at 4°C. Antibody was removed by flicking plates followed by washing 4 times with PBS-T, and 100 μl of horseradish peroxidase (HRP) conjugated avidin D (A-1004, Vector Laboratories, Burlingame, California) was added per well at 5 μg/ml in PBS-T with 1% FCS and incubated for 1 h at room temperature. Plates were washed three times with PBS-T and PBS before adding 100 μl of freshly prepared chromogen substrate. The substrate was prepared by adding 0.15 ml of AEC solution (3-amino-9-ethyl carbazole, MP Biochemical#195039) at a concentration of 20 mg/ml in dimethylformamide (Sigma, St Louis, Missouri) to 10 ml of 0.1M sodium acetate buffer (pH = 4.8). This solution was filtered through a 0.2 μm membrane and 100 μl of 3% H_2_O_2_ was added immediately before use. Plates were incubated with substrate for 8 min until spots appeared and the reaction was stopped by rinsing plates with water. Plates were allowed to dry and spots were counted.

### Quantification of LCMV titers

Viral titers were quantified as described previously.^25^ In brief, 5 × 10^5^ Vero E6 cells were plated onto each well in 6-well plates, and after 24–48 h when they reached approximately 95% confluence, the media were removed and 200 μl of serial dilutions (of viral stock or tissue homogenates) were added dropwise on top of the monolayer of the cells. Plates were rocked every 10 minutes in a 37°C, 5% CO_2_ incubator for 1 h. Two hundred microliters of medium were aspirated out, and the monolayers were gently overlaid with a 1:1 mixture of 2 × 199 medium (20% FBS, 2% penicillin/streptomycin, 2% l-glutamine) and 1% agarose at 37°C. After 4 days, a second overlay was added, consisting of a 1:1 solution of 2 × 199 medium, 1% agarose, and 1:50 of neutral red. The overlay was removed on day 5, and plaques were counted using a conventional light microscope.

### Statistical analysis

Statistical tests used are indicated on each figure legend. In the data, horizontal shaded lines in the figures represent the limit of detection. Data were analyzed using Prism version 9 (Graphpad).

## Data Availability

These data will be provided upon request to the senior author.
